# Functional redundancy enhances microbial resilience in streams: mitigating flow perturbations

**DOI:** 10.3389/fmicb.2025.1581882

**Published:** 2025-11-28

**Authors:** Qiaoyan Lin, Yixin Zhang, Rob Marrs, Naicheng Wu, Raju Sekar, Noël Juvigny-Khenafou, Christoph D. Matthaei, Jeremy Piggott

**Affiliations:** 1 The XIPU Institution, Xi'an Jiaotong-Liverpool University, Suzhou, Jiangsu, China; 2 International One Health Institute, Wenzhou-Kean University, Wenzhou, Zhejiang, China; 3 Department of Environmental & Sustainability Science, The Dorothy and George Hennings College of Science, Mathematics and Technology, Kean University, Union, NJ, United States; 4 School of Environmental Sciences, University of Liverpool, Liverpool, United Kingdom; 5 Department of Geography and Spatial Information Techniques, Center for Land and Marine Spatial Utilization and Governance Research, Ningbo University, Ningbo, Zhejiang, China; 6 Department of Biosciences and Bioinformatics, School of Science, Xi'an Jiaotong-Liverpool University, Suzhou, China; 7 Institute for Environmental Sciences (IES), University of Koblenz-Landau, Landau, Germany; 8 Department of Zoology, University of Otago, Dunedin, New Zealand; 9 Trinity Centre for the Environment & Department of Zoology, School of Natural Sciences, Trinity College Dublin, The University of Dublin, Dublin, Ireland

**Keywords:** climate change, anthropogenic disturbance, flow intermittence, habitat heterogeneity, microbial network, ecological resilience

## Abstract

Climate-change-induced and anthropogenic flow intermittency and habitat reduction threaten freshwater biodiversity and ecosystem functioning. Stream ecosystems are increasingly being evaluated for their capacity to endure climate change and anthropogenic disturbances. It remains uncertain how stream ecosystems can withstand multiple disturbances caused by habitat degradation and increasing flow intermittency. We conducted a mesocosm experiment in an ExStream system using benthic biofilm bacteria as a bioindicator to test microbial resilience to drying perturbations, followed by rewetting in streams of different habitats relative to continuous flow. The bacterial communities were compared in three types of habitat heterogeneity and two types of drying perturbation. We investigated how habitat heterogeneity influences bacterial community composition, microbial ecological networks, and ecosystem functioning under drying conditions and recovery after rewetting. The bacterial community composition shifted after drying events and flow resumption. Long-term drying led to decreased bacterial richness but increased bacterial diversity, measured by the Shannon index. Drying networks displayed greater complexity and vulnerability than control networks. These patterns were mitigated by flow resumption, resulting in comparable α-diversity and reduced microbial network complexity and vulnerability compared to the untreated controls. Long-term drying enabled bacterial survival by forming cysts but shifted microbial functions, with reduced xylan degraders, nitrogen fixers, ammonia oxidizers, and improved chitin degraders and atrazine metabolizers in diverse-heterogeneity habitats. Upon rewetting, microbes were rapidly activated and recolonized, and there was an increase in microbial metabolism processes, i.e., chitin degraders and aromatic hydrocarbon degraders. Despite variations in species composition across different stream habitats, hydrological connectivity and functionally analogous species supported by a complex microbial network contributed to the resilience and stability of benthic bacteria against environmental disturbances.

## 1 Introduction

Climate change and anthropogenic disturbance-induced habitat degradation threaten freshwater biodiversity and the ecosystem function, which in turn influence aquatic ecosystems' stability and security (Brauer and Beheregaray, 2020; Dudgeon et al., 2006). The integrity and adaptability of stream ecosystems are being increasingly evaluated for their capacity to endure multiple disturbances in aquatic ecosystems (Dzubakova et al., 2018). Some studies have demonstrated the capacity of certain stream ecosystems to rebound following habitat degradation, attributed to factors such as the presence of refugia and recolonization events (Bǎnǎduc et al., 2021; Doretto et al., 2020). It remains unclear about the coupling of substrate habitat heterogeneity, ecological processes, and the recovery dynamics of benthic communities to anthropogenic disturbances. Additionally, most research has focused on the direct impacts of individual disturbance, such as habitat degradation or flow disturbance; the cumulative and synergistic effects of multiple stressors on freshwater ecosystems have been less studied (Morris et al., 2022). It is, therefore, essential to know if a stream with a heterogeneous substrate habitat is resistant to environmental stress, such as intermittent flows induced by changing climate or rapidly increasing human activities (Datry et al., 2017).

With the increasing intensity of global warming and damming, the variability in water flow may lead to extended droughts, streams and rivers in many regions worldwide may shift from perennial to intermittent flow regimes (e.g., Mediterranean streams) (Döll and Schmied, 2012). Flow intermittency severely impacts ecosystem structure, functioning, and fluvial systems' integrity and stability (Sharma and Goyal, 2018; Sutherland et al., 2008). Intermittent flows disrupt hydrological connectivity, affect durations and volumes of surface and hyporheic flows, and shift habitat patches within channels, all of which lead to continuous fluctuations in lotic ecological processes and resident benthic communities (Datry et al., 2016; Dudgeon et al., 2006). Specifically, prolonged drought threatens the integrity, functioning, and community composition of sediment-associated biofilms (Amalfitano et al., 2008; Gionchetta et al., 2020).

Biofilms are complex assemblages of surface-attached microorganisms, considered valuable bioindicators for evaluating environmental health, as they play an essential role at the base of aquatic food webs (Lear et al., 2012). They are also crucial for biogeochemical processes and ecosystem function, such as nutrient utilization and water self-purification (Battin et al., 2003; Zeglin, 2015). Biofilms can also be critical in regulating critical ecological processes in porous ecosystems associated with habitat heterogeneity (Scheidweiler et al., 2019). They are known to be sensitive to several environmental factors, including substrate characteristics, flow rates, nutrient concentrations, and contaminants (Lear et al., 2008; Timoner et al., 2020). The impact of flow intermittency on benthic biofilms depends on a combination of environmental conditions and biological factors (Coulson et al., 2022; Sabater et al., 2016). Biofilm bacteria interact in complex ways that can form microbial networks, the complexity and stability of these networks are essential for maintaining community stability and resilience toward environmental perturbations such as drying or flow changes (Busi et al., 2024). However, whether streams with reduced in-stream habitat heterogeneity can support the resistance of biofilm bacterial communities under drying conditions remains unknown.

Resilience can be defined as the capability of a community to withstand perturbations and maintain its distinctness in structure and identity by retaining fundamental functions (Mori et al., 2013). The ecological resilience of aquatic communities has been shown to depend on *inter alia* resource competition/facilitation, recruitment, and habitat heterogeneity (Van Looy et al., 2019). To benthic microbial communities, it has been suggested that habitat-specific substrate characteristics may dictate the microbial community, and the speed of dispersal of microbial communities throughout stream networks may aid the resilience of biofilms toward drought perturbation (Hempel et al., 2010; Székely and Langenheder, 2017). Microbial biofilms exhibit a range of adaptive strategies to endure desiccation and survive in dry environments, including the formation of specialized structures such as microbial mats, resting cysts, and thickened cell walls (Philippot et al., 2021; Romaní et al., 2013). Empirical evidence for the habitat-specific resilience of biofilm microbial communities to flow perturbations is limited (Johnstone et al., 2016).

To help address this knowledge gap and propose effective management and conservation strategies for maintaining the integrity and functioning of freshwater ecosystems, we investigated the resilience of biofilm bacterial communities to drying perturbations using a stream mesocosm experiment where we compared different levels of habitat heterogeneities (low, medium, and high) and flow regimes (control and drying). We investigated the following questions and tested the corresponding hypotheses:

(i) How does habitat heterogeneity affect benthic bacterial community composition and diversity? We hypothesize that increasing habitat heterogeneity would support a more diverse bacterial community (Zeglin, 2015).
(ii) How does the benthic bacterial community shift under different flow regimes? We hypothesize that the bacterial community composition will change under drought conditions (Pohlon et al., 2013). All streams undergoing drought conditions would have a decreased bacterial richness due to the shortage of supportive resources. Drying conditions create a finer-mosaic habitat, consequently fostering an increase in bacterial diversity within drought mesocosms (Timoner et al., 2014; Datry et al., 2017).
(iii) How do microbial functions evolve with drying and habitat degradation? We hypothesize that environmental stressors, such as changes in water flow, can significantly impact the metabolic functions of bacterial communities. This is likely due to a shift in these communities toward taxa that are better adapted to terrestrial conditions found in drying streams (Freixa et al., 2024).
(iv) How resilient are the benthic bacteria in streams with different habitat heterogeneity? We hypothesize that freshwater bacteria have a high degree of resilience toward drought after the flow resumes through microbial reactivation and recolonization (Bogan et al., 2017). Mesocosms with more heterogeneous substrate habitats should provide more refuges for organisms encountering drying conditions (Romaní et al., 2017), thus exhibiting higher drought resilience than less varied habitats.

## 2 Materials and methods

### 2.1 Experimental set-up

**Figure 1:**
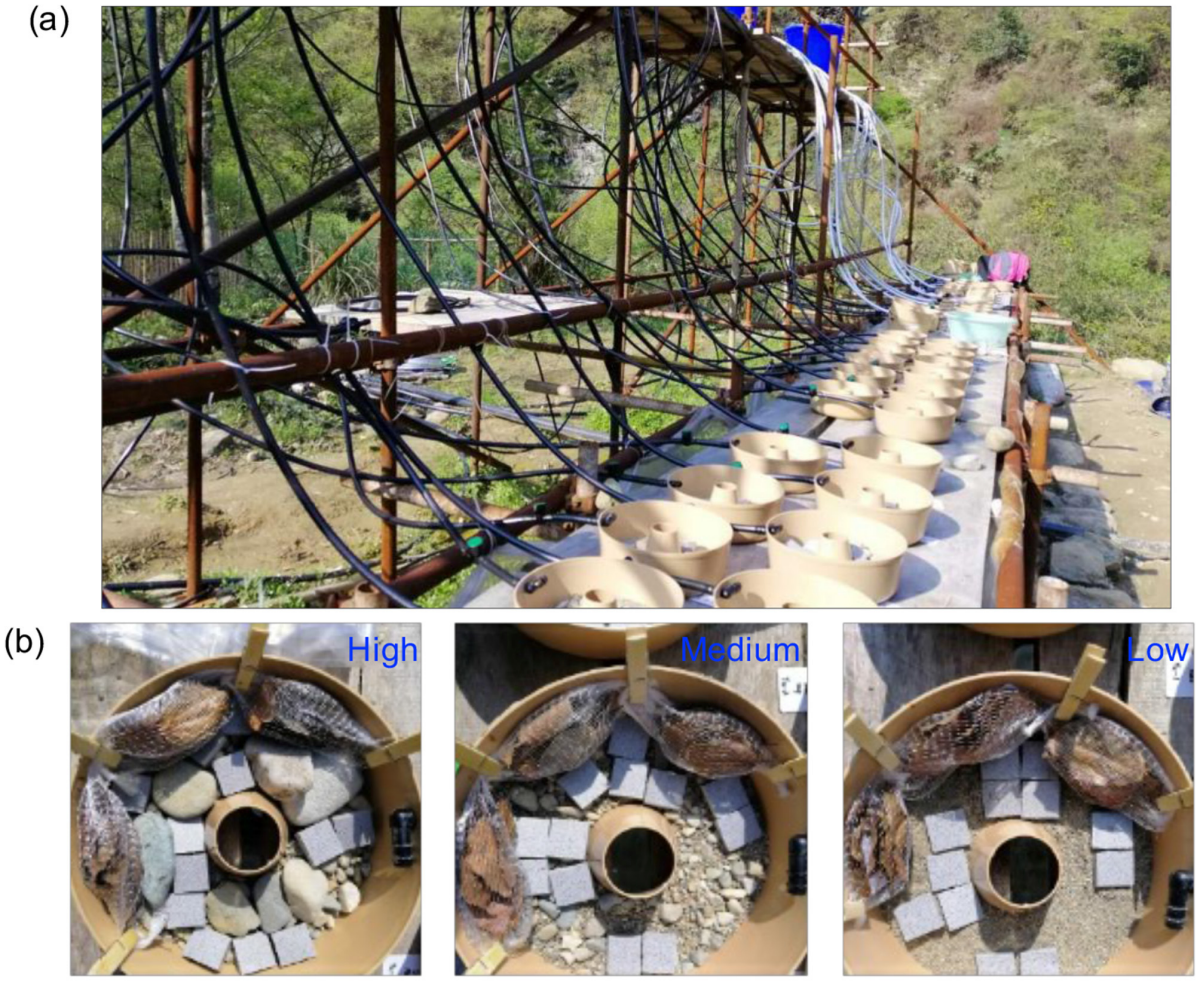
**(a)** The ExStream System mesocosm set-up on the bank of the Yinxi stream located outside the Huangshan Jiulongfeng Nature Reserve in China, and **(b)** the three substrate heterogeneity treatments (High, Medium, Low).

Our study was performed in spring (7^th^ April to 6^th^ June 2019) on the bank of the Yinxi Stream, a near-pristine second-order montane stream, outside the Huangshan Jiulongfeng Nature Reserve (Anhui province, China, 30°7′07^′′^N, 118°1′24^′′^E). The experiment used a stream mesocosm set-up (ExStream System; Piggott et al., 2015). The ExStream System (ExStream Systems Ltd., Dunedin, New Zealand; Figure 1) is a tightly controlled, statistically powerful, yet highly field-realistic set-up, resulting in the publication of over 30 articles. A detailed description of the Yinxi Stream set is presented in Juvigny-Khenafou et al. (2020). This study used a modified version, with four header tanks feeding 12 channels each. The mesocosms (Microwave Ring Molds, Interworld, Auckland, New Zealand; external diameter 24.5 cm, inner diameter 5.1 cm, volume 3 L) were supplied continuously with water pumped from the Yinxi Stream into four header tanks through a 4-mm mesh filter, and then directed to each stream mesocosm, enabling natural immigration and emigration of stream periphyton, microbes, and invertebrates by drift and egg deposition.

### 2.2 Experimental design

A BACI (Before-After-Control-Impact, Smith, 2002) design was adopted (Supplementary Figure S1). There were three substrate habitat treatments: (1) Sediment, (2) Sediment+Gravels, and (2) Sediment+Gravels+Pebbles+Cobbles representing three levels of in-stream habitat heterogeneity and henceforth denoted Low, Medium, and High heterogeneity, respectively (Mora-Gómez et al., 2016), fully crossed with two flow treatments: normal flow (continuous flow throughout the experiment) vs. drying (15 days without flow followed by 23-day rewetting), denoted Control and Drying, respectively. This provided six treatment combinations, each with eight replicates. All replicates of each treatment combination were assigned randomly to the mesocosms in a randomized block design with four spatial blocks and two replicates of each treatment combination per block.

Habitat heterogeneity treatments were implemented at the start of the experiment before the water flow began. The Low-heterogeneity treatment comprised 700 mL of fine sediment (< 2 mm). The Medium-heterogeneity treatment consisted of 300 mL fine sediment (< 2 mm) and 400 mL gravel (2–30 mm in diameter). The High heterogeneity treatment comprised 300 mL fine sediment (< 2 mm), 900 g gravels (2–30 mm in diameter), four pebbles (30–64 mm), and three cobbles (>64 mm length). All substrata were collected from the Yinxi Stream and air-dried for 72 h before addition to the mesocosms. Twelve autoclaved ceramic tiles (dimensions 3.0 × 3.0 × 1.0 cm) were placed randomly on the bed surface of each mesocosm as standardized substrates for biofilm colonization. Three leaf-litter bags (8 mm mesh size) containing 2.50 ± 0.005 g of dried camphor tree (*Cinnamomum camphora* L.) leaves were added to each mesocosm before water flow started to provide an additional food source and habitat for stream biota. Leaf bags were clipped to the side of each mesocosm and laid on the substratum, similar to field conditions where leaf packs form in streams. Apart from the drought period in the Drying treatment, water discharge through each mesocosm was calibrated daily and maintained at 2 Lmin^−1^, which resulted in a near-bed flow velocity of 12 cms^−1^. Flow velocity was measured weekly in all mesocosms using an electromagnetic flow meter (MF Pro, OTT HydroMet GmbH, Germany).

The experiment ran for 61 days (Supplementary Figure S1). After 23 days of natural colonization with continuous flow through all mesocosms, the water supply of the Drying treatment was shut off for 15 days (with two rounds of biofilm sampling after 3 days and 15 days of drought), followed by 23 days of rewetting. To simulate the actual water loss of drought, large amounts of water were pumped out from the drying treatment mesocosms after the start of the drought so that the upper layer of the substrate was covered by 2 cm of water; this was allowed to air dry. The 3-day de-watering period was long enough to drain the surface water from the mesocosms but still short enough to prevent significant changes in the physico-chemical conditions inside the substrate, thus retaining substrate availability as a drought refuge (Ledger et al., 2011). The 15-day de-watering period is based on the experiment of McKew et al. (2011).

### 2.3 Biofilm sampling procedure

On each of the four dates (after 23 days of colonization, 3 days of drought, 15 days of drought, and 23 days of rewetting), three ceramic tiles were randomly selected from each mesocosm for biofilm analysis. Biofilm samples from each mesocosm were obtained by scraping the accumulated periphyton material from the upper surfaces of these three tiles into 50 mL tubes using a toothbrush with shortened bristles, including wet biofilm samples taken from flowing streams, and drought samples collected from drought mesocosms after 3 days of drought and after 15 days of drought. These tubes were then covered with aluminum foil for transport on ice in the dark to the laboratory within 4 h. In the laboratory, these samples were filtered through 0.22-μm pore size polycarbonate membrane filters (Millipore, Boston, MA, USA) using a vacuum pump and stored in sterile Petri dishes at −20 °C until DNA extraction.

### 2.4 Bacterial community composition analysis

Based on the manufacturer's protocol, 250 mg of the biofilm samples was used for DNA extraction using the Mag-Bind Soil DNA Kit (Omega E.Z.N.A.TM, Omega Bio-Tek, Norcross, GA, USA). After that, using 10–20 ng DNA as the template, the V3-V4 region of bacterial 16S rRNAs was amplified using the PCR primer pairs (forward/reverse) 341F/805R provided by Sangon Biotech (Shanghai) Co., Ltd. (Lin et al., 2019). A Nested PCR was later performed using 20 ng of the previous PCR products. The PCR-amplified products were checked by agarose gel-electrophoresis to confirm amplification success, ensure specificity, and estimate product size, then quantified using the Qubit 3.0 DNA test kit (Life Technologies, Carlsbad, CA, USA), and purified with Agencourt AMPure XP beads (Beckman, Brea, CA, USA). Amplicons were pooled in equal concentrations in the final mixture. The final sequencing concentration was set at 20 pmolmL^−1^ using 10 ng of DNA per sample. All samples were subjected to paired-end sequencing with a 2 × 300 bp read length.

Sequences were processed and analyzed via QIIME 1.8.0. Following primer removal, all low-quality reads containing ambiguous characters, a sequence length less than 200 bp, and a tail quality score < 20 were discarded. Chimeras were assessed using the UCHIME software and discarded. The remaining high-quality reads were clustered into OTUs (Operational Taxonomic Units) via USEARCH with a 97% similarity (Edgar, 2010). OTUs observed only in one read across the dataset were removed. The average length of the remaining filtered sequences was 412 bp. All OTUs were assigned to the taxonomic category against the Silva132 database using the Ribosomal Database Project classifier (RDP Version 2.12) at a confidence threshold of 0.8.

### 2.5 Statistical analysis

The α-diversity indices (Shannon-Wiener index; bacterial taxon richness) of the OTUs were calculated in QIIME 1.8.0. After that, most of the statistical analysis was performed using SPSS Statistics software v26; the “vegan” package in the R statistical environment (Oksanen et al., 2018) was used for NMDS and ANOSIM analysis (R Core Team, 2017).

The statistical model structure used for total bacterial analysis was the following: intercept (d.f. 6) + habitat (1) + hydrology (1) + time (1) + habitat ^*^ time (1) + hydrology ^*^ time (1) + habitat ^*^ hydrology (1) + habitat ^*^ hydrology ^*^ time (1) + error (168; n = 192), while the two-way MANOVA model structure applied for specific experiment phases was as follows: intercept (d.f. 2) + habitat (1) + hydrology (1) + habitat ^*^ hydrology (1) + error (42; n = 48). The linear model detailed above analyzed differences in bacterial α-diversity indices (bacterial taxon richness, Shannon-Wiener diversity) across the habitat heterogeneity and drying treatments. A 2-way MANOVA with habitat heterogeneity and hydrology as two independent variables was also used, and least significant difference (LSD) *post-hoc* tests calculated the 14 most abundant bacterial phyla of different phases in cases with significant overall differences (>1% of the total reads; Baltar et al., 2015). Non-metric Multi-dimensional Scaling (NMDS) was then applied to examine bacterial β-diversity based on Euclidean dissimilarities. Analysis of similarities (ANOSIM) was conducted based on Bray-Curtis distances to assess the similarity of bacterial community composition (including rare taxa) across the habitat heterogeneity and drying treatments on the four sampling dates. Bonferroni correction was used to adjust the significance level for multiple comparisons to control the overall type I error rate.

Thereafter, co-occurrence networks were constructed based on the Spearman correlations (ρ > 0.7, *p* < 0.001) of bacterial OTU abundances (Yuan et al., 2021). The topological indices of microbial networks were calculated, including the number of nodes, number of edges, connectance, average degree (AD), average clustering coefficient (avgCC), and graph density (GD). The within-module connectivity (Z) and among-module connectivity (P) for each node were calculated to classify its topological role within the overall network. Vertices with high Z- or P-scores were identified as keystone taxa, including peripherals (Zi < 2.5, Pi < 0.62), connectors (Zi < 2.5, Pi > 0.62), network hubs (Zi > 2.5, Pi > 0.62), and module hubs (Zi > 2.5, Pi < 0.62) (Banerjee et al., 2018). The empirical networks were constructed based on the power-law model using the “igraph” package in R and then visualized using the Gephi platform (http://gephi.github.io/). Moreover, network complexity metrics (e.g., network complexity, robustness, and vulnerability) were analyzed according to Zhang et al. (2024) to assess the stability and resilience of microbial co-occurrence patterns. A higher network complexity score indicates a more balanced network structure with a greater potential to maintain stability under environmental changes. Network robustness reflects the proportion of remaining species after the random removal of certain parts of the species (Montesinos-Navarro et al., 2017). Network vulnerability represents a network's susceptibility to disruptions caused by node or edge removal (Deng et al., 2012).

Finally, the METAGENassist web server was used to evaluate shifts in the functional structures of bacterial communities under drought conditions and after rewetting (Arndt et al., 2012). Twenty-nine metabolic functional categories were examined to assess the biochemical processes of these communities. A two-way MANOVA was performed on the abundant functions (>1% of the total reads, Baltar et al., 2015) to investigate specific functional responses, taking habitat heterogeneity and hydrology as independent factors. Differences were considered significant at *p* < 0.05. Standardized effect sizes (Partial eta-squared, range 0–1) are presented for *p*-values < 0.1 to allow interpreting the biological relevance of the findings (Garson, 2015). Effect sizes are classified as follows: < 0.01 very small, ≥0.01 small, ≥0.06 medium, and ≥0.14 large (Richardson, 2011).

## 3 Results

### 3.1 α-diversity metrics

The dataset had 5,529,461 raw reads from the 192 samples. After filtering, denoising, and chimera removal, 5,146,771 reads were assigned to 78,100 OTUs. Overall, all samples' bacterial taxon richness and Shannon-Wiener diversity changed significantly through time and hydrology changes (Supplementary Table S1). Bacterial richness and Shannon-Wiener diversity at 23d and 26d are similar, but these values were distinct from those at 38d and 61d under continuous flowing (Supplementary Table S1c). Hydrology changes altered the Shannon-Wiener diversity through time. Bacterial richness declined dramatically under hydrology changes, while Shannon-Wiener diversity showed an opposite pattern (Supplementary Table S1a). Habitat heterogeneity markedly changed the bacterial richness. With increased habitat complexity, mesocosms with high heterogeneity had lower bacterial richness and a slightly decreased diversity of bacteria than low-heterogeneity ones (*p* = 0.009, *p* = 0.064, respectively). No interaction effect between habitat and hydrology was observed on both metrics, along with time changes.

**Figure 2:**
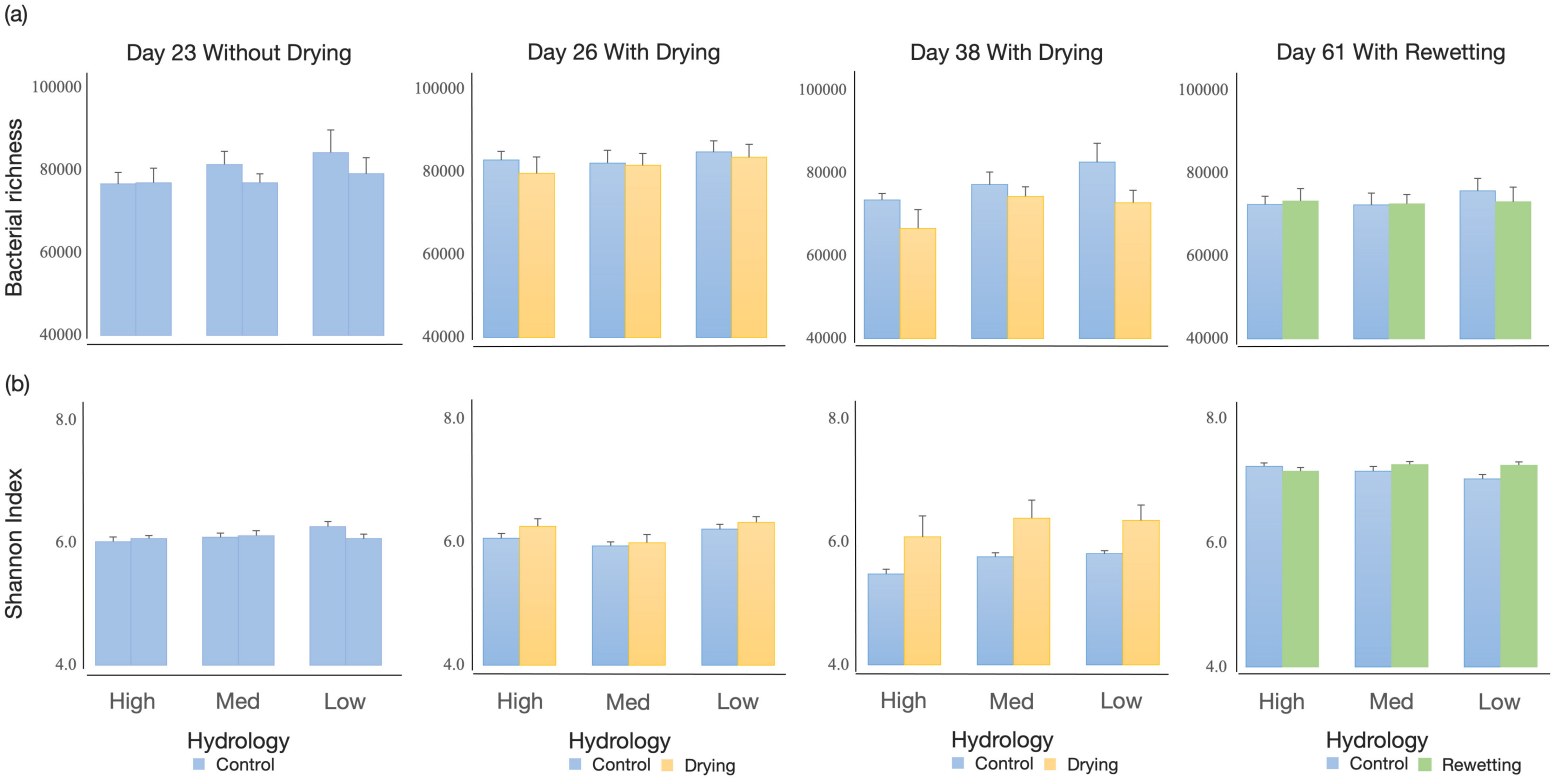
Effects of different flow conditions on **(a)** bacterial richness and **(b)** Shannon index through time (Day 23, Day 26, Day 38, Day 61) in a mesocosm experiment. The three flow conditions were (1) continuous flowing (blue), (2) a Drought period (yellow), followed by (3) rewetting (green), all tested stream substrates with High, Medium, and Low heterogeneities. Mean values (+SE) are presented.

After the initial 23-day colonization, habitat heterogeneity did not affect α-diversity indices (Supplementary Table S2 and Figure 2). After 3-day drying, habitat heterogeneity significantly affected the Shannon-Wiener diversity, where both low and high habitat heterogeneity treatments had more diverse bacteria than medium-heterogeneity ones (*p* = 0.004, *p* = 0.053, respectively). Hydrology treatment did not affect either α-diversity metric. After 15-day drying, habitat heterogeneity slightly affected bacterial richness. The mesocosms with low and medium habitat heterogeneity had greater bacterial richness than high-heterogeneity ones (*p* = 0.026, *p* = 0.094, respectively). Hydrology had a distinct impact on α-diversity indices, with the drying mesocosms having a lower bacterial richness but a greater Shannon-Wiener diversity than the continuous flow treatment (Supplementary Table S2). After flow resumed, no differences in α-diversity indices across habitat treatments were detected, except for a slight increase in bacterial diversity in rewetting mesocosms (Supplementary Table S2). Bacterial diversity was strongly affected by the interaction between habitat and hydrology. Shannon-Wiener diversity increased strongly in rewetting mesocosms with low heterogeneity.

### 3.2 Bacterial community composition

**Figure 3:**
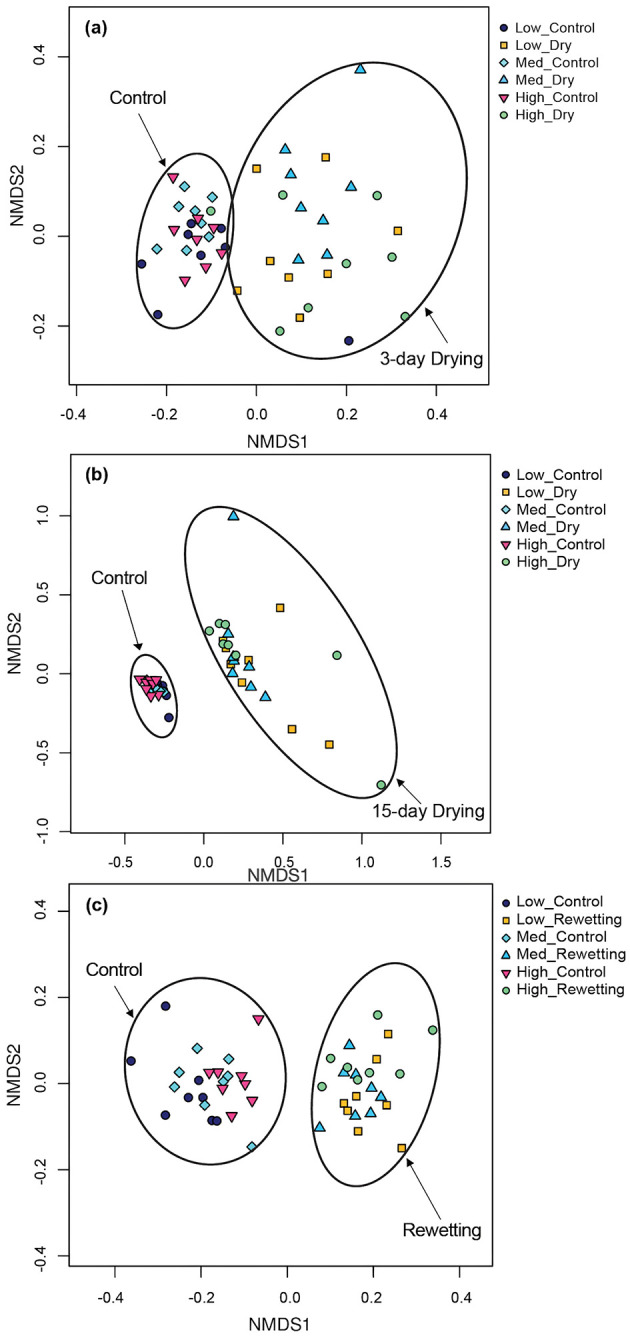
Ordination (non-metric multi-dimensional scaling) of biofilm bacterial communities in a mesocosm study under continuous flowing (Control) and drought perturbation (Drying/Rewetting) conditions after **(a)** 3 days drying, **(b)** 15 days drying, and **(c)** rewetting in high, medium, and low heterogeneous habitat streams. The ellipses were drawn by hand in the figures to assist in visualizing the data distribution and groupings.

After Bonferroni correction, the overall bacterial community structure was similar across the three habitat heterogeneity treatments at the end of the 23-day, 26-day, 38-day, and 61-day colonization period (Anosim, Supplementary Table S3a). No significant differences in bacterial community were detected among mesocosms with different levels of habitat heterogeneity after being subjected to three-day drying, 15-day drying, and rewetting (Supplementary Table S3b). Community composition under drying conditions differed from that under permanent flow (Figures 3a–c and Supplementary Table S3c) after 3 or 15 days of drying. The same was still the case after rewetting (Supplementary Table S3c). In the NMDS, bacterial communities under drying conditions were separated along the first axis on all three post-drying dates (Figure 3).

### 3.3 The most abundant bacterial phyla

**Figure 4:**
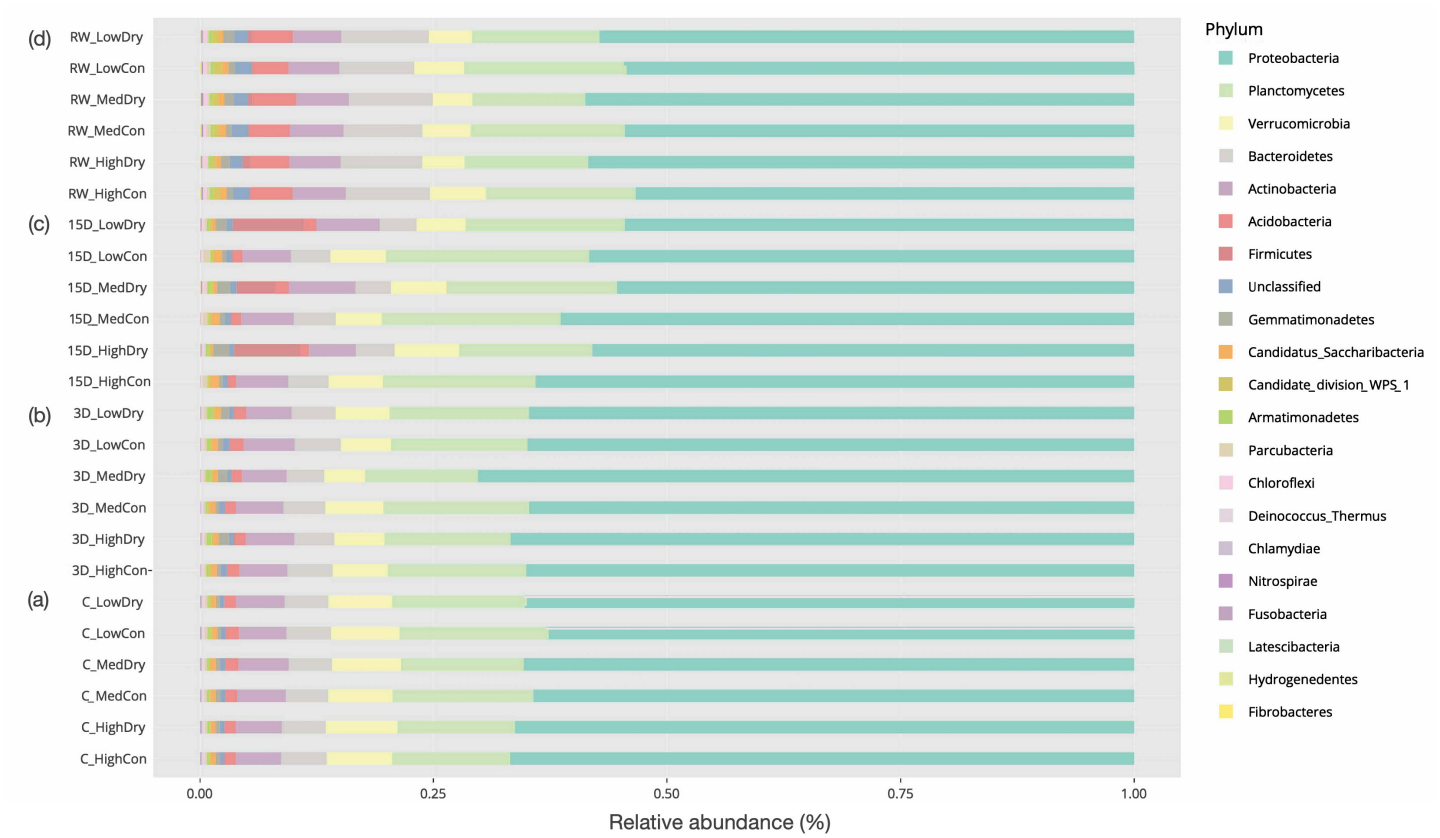
Mean relative abundances of bacterial Phyla under **(a)** continuous flowing (Control) and after **(b)** 3-day drying, **(c)** 15-day drying, and **(d)** rewetting in high, medium, and low heterogeneous habitat streams.

The bacterial community was predominantly composed of Proteobacteria (66.8% of all reads), along with Planctomycetes (12.63%), Verrucomicrobia (7.01%), Bacteroidetes (4.88%), Actinobacteria (4.85%), and other phyla, which constitute the most abundant taxa (Figure 4). The 14 most abundant bacterial phyla observed in all mesocosms were affected by hydrology, experiment duration, as a main effect, and the interaction between hydrology and experiment duration (Supplementary Table S1b)−28.6% for enhanced habitat heterogeneity, 57.1% for hydrology changes, 92.9% for experiment phases. Regarding stressor interactions, 57.1% were affected by hydrology along with experiment duration, 14.3% for habitat and research duration, and no significant interaction was observed between habitat and hydrology (Supplementary Table S1b).

Specifically, the drying treatment significantly affected the most abundant phyla after 15-day drying and 23-day rewetting (Supplementary Table S4). After 3-day drying, 28.6% of bacterial taxa (positive: Gammatimonadetes, Armatimonadetes, Firmicutes; negative: Candidatus Sacchari bacteria) were affected, 21.4% for the improved habitat (positive: Acidobacteria; negative: Bacteroidetes, Parcinacteria). The medium-complexity treatment had less Bacteroidetes and Acidobacteria abundance than the low ones (*p* = 0.015, *p* = 0.016, respectively). After 38 days, 64.3% of taxa (positive: Gemmatimonadetes, Acidobacteria, Armatimonadetes, Firmicutes, Chloroflexi; negative: Proteobacteria, Planctomycetes, Candidatus Sacchari bacteria, Parcubacteria) were affected by 15 days of drying, 28.6% for the improved habitat (positive: Acidobacteria, Candidate division WPS-1; negative: Planctomycetes, Chloroflexi). The high-heterogeneity treatment possessed fewer Planctomycetes, Acidobacteria, Candidate division WPS-1, and Chloroflexi than the low-heterogeneity ones (*p* = 0.011, *p* = 0.013, *p* = 0.019, *p* = 0.010, respectively). The abundance of Actinobacteria, Acidobacteria, Candidate division WPS-1, and Chloroflexi was greater in medium-heterogeneity treatment than in the high-heterogeneity ones (*p* = 0.046, *p* = 0.013, *p* = 0.011, *p* = 0.018, respectively). Rewetting impacted 57.1% of taxa (positive: Gemmatimonadetes, Firmicutes, Chloroflexi, Deinococcus-Thermus; negative: Planctomycetes, Verrucomicrobia, Candidatus Sacchari bacteria, Parcubacteria). Meanwhile, 7.1% of taxa showed significant effects for improved habitat (negative: Gemmatimonadetes) and 7.1% for the interaction of habitat and hydrology changes (negative: Firmicutes). After flow resumed, the low-heterogeneity treatment had a greater abundance of Gemmatimonadetes than the high and medium ones (*p* = 0.008, *p* = 0.037, respectively). In contrast, the abundance of Deinococcus-Thermus was greater in the high-heterogeneity treatment than in the medium-heterogeneity treatment (*p* = 0.042).

### 3.4 Microbial networks

**Figure 5:**
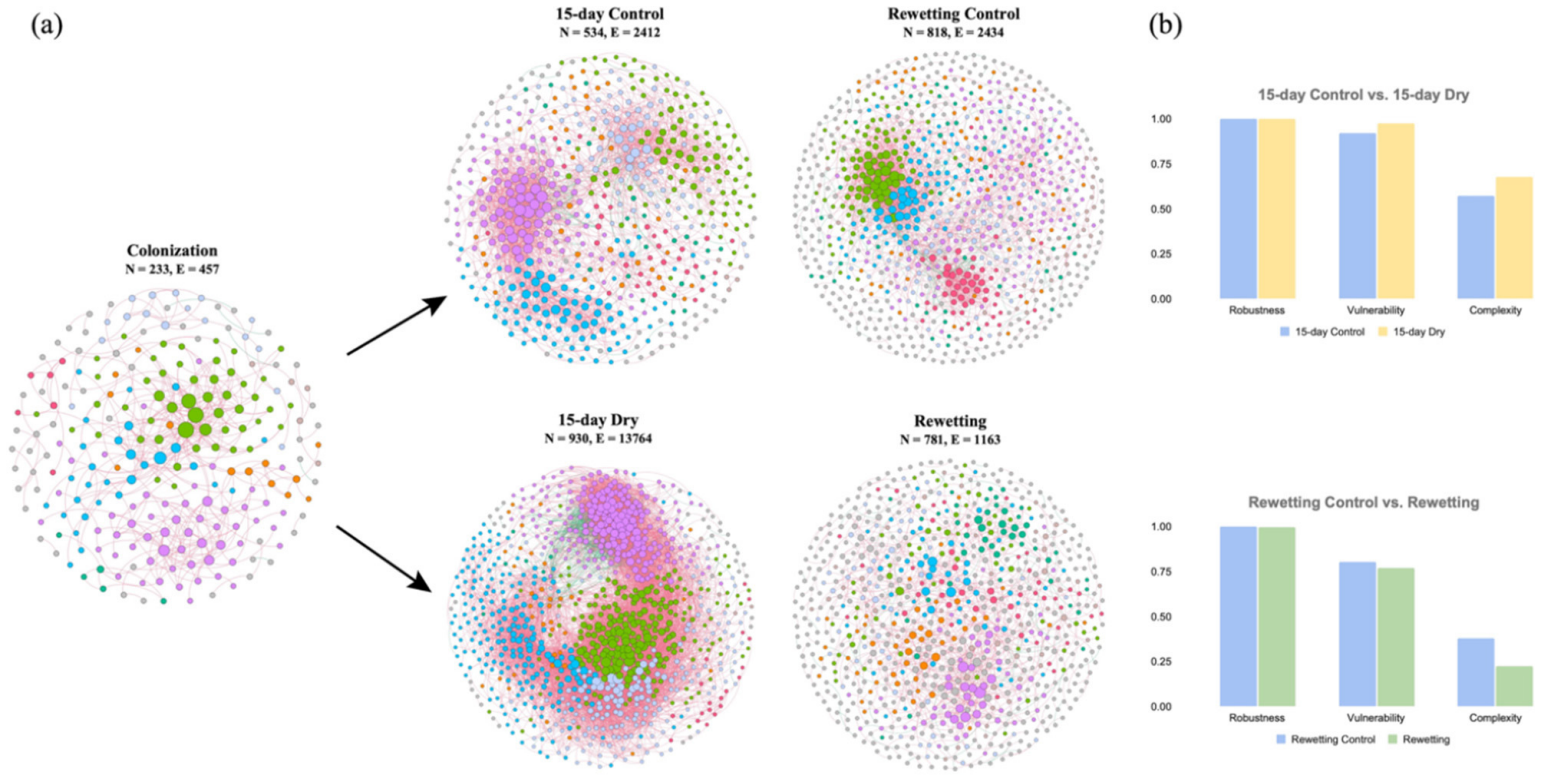
**(a)** The bacterial co-occurrence network constructed by core OTUs. Eight dominant modules were presented in different colors in each network. Red lines represent positive relationships in relative abundances, while green lines indicate negative relationships. **(b)** Microbial network complexity and stability under drying and flow resumption.

Microbial co-occurrence networks were created for bacteria under 23-day colonization, followed by 15 days of drying and subsequent rewetting, consisting of 3,298 nodes and 20,230 edges. These networks displayed enhanced complexity with long-term drying and decreased complexity after flow resumption, characterized by an increase in nodes and edges under 15-day drying and a decrease of these metrics thereafter rewetting (Figure 5a). Similarly, drying networks exhibited greater vulnerability than control ones, while rewetted networks showed the opposite trend (Figure 5b). These networks predominantly featured positive associations, accounting for over 92% of all bacterial networks (Supplementary Table S6). Further, various topological features, including connectance, AD, avgCC, and GD, consistently increased after 15 days of drought perturbation and then decreased when normal flow resumed.

Bacterial networks displayed a great degree of modularity, with over 50% of nodes assigned to 8 modules (Supplementary Table S5). The module nodes and connectors identified as keystone species for structuring the network were evaluated based on their within-module connectivity (Zi) and among-module connectivity (Pi). A total of 76 module hubs and 60 connectors were identified across all networks constructed (Supplementary Table S5). During early colonization, 10 of the 25 keystone nodes were Planctomycetes, while 7 belonged to Proteobacteria. Planctomycetes and Proteobacteria account for 40% and 36.7% of the keystone species under 15-day control, compared to 31.25% of Proteobacteria, 25% of Bacteroidetes, 25% of Planctomycetes, and 15.6% of Actinobacteria after 15-day drying. Upon flow resumption, Proteobacteria, Bacteroidetes, and Planctomycetes accounted for 37.5%, 29.17%, and 16.7% of the keynote species, respectively. This is compared to 36% of Proteobacteria and 28% of Planctomycetes during continuous flow in the rewetting period. Proteobacteria, Planctomycetes, and Bacteroidetes associated keynote species were detected in all bacterial networks, indicating a significant co-occurrence.

### 3.5 Bacterial metabolic functions

The Metagenassist analysis generated 29 predicted bacterial metabolic functions, eleven abundant metabolic functions were distributed in all treatments (Supplementary Table S6), constituting 97.7% of all functions identified.

Three-day drying remarkably decreased 27.3% of the abundant metabolic functions (Dehalogenation, Xylan degrader, and Sulfur oxidizer) and slightly increased the nitrite reducer. 9.1% showed a main effect for the improved habitat (Supplementary Table S6). The medium-heterogeneity and high-heterogeneity treatments had reduced aromatic hydrocarbon degraders compared to those with low-heterogeneity (*p* = 0.019, *p* = 0.009, respectively). After 15-day drying, 36.4% of the abundant metabolic functions showed a main effect for drought perturbation (positive: Chitin degrader, Atrazine metabolizer; negative: Sulfate reducer, Nitrogen fixer), the ammonia oxidizer and xylan degrader were also slightly decreased with a drying effect. Additionally, 27.3% showed a main response to enhanced habitat heterogeneity (negative: Xylan degrader, Aromatic hydrocarbon degrader, and Sulfur oxidizer). High-heterogeneity treatment possessed fewer xylan degraders, aromatic hydrocarbon degraders, and sulfur oxidizers than low-heterogeneity ones (*p* = 0.004, *p* = 0.006, *p* = 0.004, respectively). Medium-heterogeneity mesocosms had a greater xylan degrader than high-heterogeneity ones and had fewer sulfur oxidizers than the low-heterogeneity ones (*p* = 0.035, *p* = 0.045, respectively). After flow resumed, 18.2% of abundant metabolic functions were affected by hydrology changes (positive: Aromatic hydrocarbon degrader and Chitin degrader), 9.1% for the interaction between habitat improvement and hydrology changes (positive: Atrazine metabolizer). No difference was detected among the three habitat types for any metabolic function.

## 4 Discussion

### 4.1 Increasing habitat heterogeneity would not shift the bacterial community

Our results indicate that habitat change did not lead to a difference in the overall structural and α-diversity in bacterial communities under continuous flowing conditions throughout the experiment. Our results do not, therefore, support our first research hypothesis that a more heterogeneous habitat would support more diverse benthic bacteria. These results differ from previous research that showed a mixed substrate would provide a greater range of divided habitat spaces for colonizing diverse taxa (Giller and Malmqvist, 1998). They additionally vary from prior investigations that different habitats may foster the specialization of bacterial composition, as the contrasting micro-conditions present contrasting environments, allowing for the dominance of various microbial heterotrophic taxa (Zeglin, 2015). We suspect that physical heterogeneity is not always the main driving factor controlling benthic bacterial diversity, as indicated by Palmer et al. (2010). As revealed here, bacterial diversity was predominantly influenced by the interplay between habitat and hydrology after flow resumed. These interactions, rather than the main effects, were more critical in determining the bacterial communities (Juvigny-Khenafou et al., 2020).

Although comparable bacterial community compositions were detected among mesocosms with different habitats after drying and rewetting, 15-day drying coupled with habitat improvement induced a decrease in xylan degraders, sulfur oxidizers, and aromatic hydrocarbon degraders, alongside a reduced relative abundance of Planctomycetes and Chloroflexi. In heterogeneous habitats, the uneven distribution of nutrients and microhabitats can create isolated microenvironments, which may reduce the abundance and activity of Planctomycetes and Chloroflexi taxa (Worrich et al., 2016). Both Chloroflexi and Planctomyces are commonly found in environments where lignocellulosic biomass is degraded; Planctomyces possesses enzymes for chitin degradation, which suggests their potential role in xylan degradation (Lu et al., 2019; Kulichevskaya et al., 2019; Zhang et al., 2019). The decrease in Planctomycetes and Chloroflexi abundance makes conditions less favorable for xylan degradation. Flow resumption remediated drought impacts, resulting in comparable metabolic functions across diverse habitats. These findings support our third hypothesis that alterations in water flow and habitat heterogeneity can significantly impact the potential metabolic functions of bacterial communities.

### 4.2 Bacterial community changes under drought conditions

We showed that bacterial community composition was significantly shifted under drying conditions (either after 3-day drying or 15-day drying) in all three heterogeneity habitats. This supports our second hypothesis that drying markedly affects the bacterial community experiencing drought perturbation. Hydrological variability was highly related to the variation in bacterial community composition (Battin et al., 2016). Dry substrates, moist substrates, and substrates in flowing waters produced contrasting microbial community composition and activity (Pohlon et al., 2013; Gionchetta et al., 2020).

After 3-day drying, hydrological changes did not affect bacterial α-diversity. These results suggest that the bacterial community had a degree of functional redundancy under short-term drying (Juvigny-Khenafou et al., 2020). The remaining surface water, sequence of disconnected pools, and connected hyporheic flow after the short 3-day drying period provide refuges for surviving most bacterial communities (Bhamjee et al., 2016), allowing the maintenance of similar bacterial richness in diverse habitat streams across experimental treatments. However, streams with low and high habitat heterogeneity fostered a greater diversity of bacteria, as the fine-mosaic habitat and more diverse macro-habitats created by drought perturbation provide suitable niches for taxa with preferences for these habitats (Datry et al., 2017). This may lead to the generation of more diverse bacterial communities compared to streams with medium habitat heterogeneity.

On the other hand, 15-day drying induced a slight increase in microbial network complexity and vulnerability, with a smaller bacterial richness but a greater Shannon-Wiener diversity. A relatively long-term drought may create a finer-mosaic habitat, fostering various taxa with specific habitat preferences (Datry et al., 2014, 2017), as confirmed here. Without water as a medium for oxygen transport, the fine-mosaic habitat typically has a higher surface-to-volume ratio, which, in turn, provides more sites for microbial colonization, enhances microbial activity, and raises oxygen consumption (Dang et al., 2021). In some cases, this can result in anoxic conditions, especially in areas with increased organic matter (Dang et al., 2021; Von Schiller et al., 2017; Wang et al., 2025). The form of anoxic condition potentially hindering aerobic metabolic processes within biofilm (Medeiros et al., 2009), such as aerobic processes of xylan degradation, nitrogen fixation, and ammonia oxidation, associated with a declining relative abundance of Proteobacteria and Planctomycetes. The loss of nitrogen fixers and ammonia oxidizers can lead to reduced nitrogen fixation and nitrification activity, which may potentially cause nitrogen limitations for plant growth and other biological processes (Rajput et al., 2023; Müller et al., 2025). The decline in xylan degraders may slow down plant decomposition, reduce carbon turnover and potentially affect carbon sequestration in streams (Dutschei et al., 2023). Instead, the growth rate and activities of anaerobic bacteria that are responsible for the anaerobic process of carbohydrate metabolism (Briée et al., 2007; Timoner et al., 2014) might be accelerated with oxygen depletion, increased soil temperature, and the enrichment of organic matter and nutrients (Wang et al., 2014; Wörner and Pester, 2019), such as anaerobic process of chitin degradation and atrazine metabolism, associated with an increasing relative abundance of Acidobacteria and Firmicutes, as shown in this study. Chitin is an essential element of a cyst. The formation of cysts by bacteria involves significant changes in cell wall and cell membrane biochemistry, which might induce a decrease in metabolic activity and the survival of bacteria in harsh environmental conditions (Romaní et al., 2013). Some gram-negative bacteria, such as Rhodospirillum centenum, can form metabolically dormant cysts under conditions of desiccation (Dong and Bauer, 2015). The increase in chitin and atrazine degraders facilitates the breakdown of chitin and atrazine into simpler, less harmful compounds, thus enhancing organic matter recycling and releasing bioavailable nitrogen and carbon for the survival of other microorganisms (Wieczorek et al., 2019; Guo et al., 2024). Meanwhile, a relatively long time of drying created terrestrial habitat along dry riverbeds (Boulton et al., 2017), hindering the supply of water resources for microbes and leading to the decline of bacterial richness in diverse habitat mesocosms (Timoner et al., 2014). Water resource scarcity will impact microbes' physical and biological activities, hindering their ecological processes, including carbon degradation (Gionchetta et al., 2020). However, substrates that comprise a greater surface area in low-heterogeneity and medium-heterogeneity habitats might possess greater water-holding capacity, protecting microbial cells from drying events by forming refugia under the surface area (Van Looy et al., 2019), thus sustaining more bacteria than high-heterogeneity ones, as found here.

### 4.3 Freshwater bacteria have a high degree of resilience toward drought

The resumption of flow, driven by either rainfall or water management, rewets the stream and moves the habitat from a terrestrial to a lotic environment (Boulton et al., 2017), leading to changes in microbial community composition (Zoppini et al., 2010) and a slight decline in microbial network complexity. As the experimental system remained connected to the stream and hence had a continuous input of microbes which would have helped recolonization by biofilm bacteria, buffering the losses of the original taxon in the drying treatment. This led to a comparable bacterial richness, yet slightly greater diversity of bacteria after rewetting in all treatments, compared to the control streams. The significant recovery of bacterial α-diversity to control levels following rewetting largely supported our fourth hypothesis, suggesting that freshwater bacteria have great resilience toward drought perturbation after flow resumption, per the assumption proposed by Boulton et al. (2000). The speed active of specific autochthonic “seed-bank” microbial communities, which persist during drying periods (Zeglin et al., 2011), coupled with the recolonization of microbes dispersed from the upstream environment (Rosado et al., 2015), can mitigate the drying effects on microbes. The persistence of microbial mats formed during drying periods and the presence of associated microbes may account for the observed increased bacterial diversity in rewetted streams.

Though the α-diversity recovered to control status after rewetting, streams with different heterogeneity habitats had comparable bacterial community functions in rewetting and control streams, the bacterial community composition in three stream types was distinct from the control ones, which is in accordance with Romaní et al. (2017). The flushing of microbial mats formed by cyanobacteria and heterotrophic bacteria during prolonged drying conditions (Stanish et al., 2013) and the speed recolonization of bacteria dispersed from upstream might explain the similar functional bacterial communities in rewetting systems. The return of flow led to greater chitin degraders and aromatic hydrocarbon degraders in streams undergoing drought perturbation, associated with an increasing relative abundance of Firmicutes, Gemmatimonadetes, and Chloroflexi. These results imply that bacteria in streams reduced their metabolic activity and facilitated their survival during drying (Lebre et al., 2017; Romaní et al., 2017). Upon rewetting, an aerobic and suitable physico-chemical environment returned, which activated the mineralization of phosphorus and nitrogen by keynote taxa such as Gemmatimonas (Chee-Sanford et al., 2019). Further, it enabled an active and fast assemblage of OM-decomposing species, and induced rapid excystment of organisms through enhanced chitin degraders, and breakdown of organic pollutants through aromatic hydrocarbon degraders in streams (Verni and Rosati, 2011; Han et al., 2021), such as Firmicutes members that drive the late phases of chitin degradation (Wörner and Pester, 2019). These shifts improve bioremediation and nutrient cycling, both of which are critical for microbial communities' recovery by providing carbon and nitrogen sources, while reducing harmful effects on aquatic organisms and overall stream health (Wieczorek et al., 2019; Han et al., 2021). Moreover, no difference in metabolic function was observed among various heterogeneous habitats after flow resumed.

Despite habitat and hydrological disturbances, functional redundancy may play a critical role in maintaining the stability of stream ecosystems (Biggs et al., 2020). Streams with greater microbial complexity are often occupied by species with analogous ecological processes and functions, thus ensuring the resilience of microbes and microbe-associated ecosystem functions in the face of drought fluctuations (Louca et al., 2018). In this study, genera of Planctomycetes and Firmicutes play a role in chitin degradation (Kulichevskaya et al., 2019; Wörner and Pester, 2019). While the relative abundance of Planctomycetes significantly decreased, the Firmicutes species exhibited a similar metabolic function that enhanced chitin degradation after 15 days of drying and rewetting. Further, different habitats may evolve similar functional traits due to similar selective pressures to withstand desiccation or to rapidly utilize resources after flow resumption (Martiny et al., 2023). Moreover, in line with Rolls et al. (2012) and Van Looy et al. (2019), this study revealed that stream hydrological connectivity is fundamental for the recolonization of microbial communities, heterogeneous substrates within stream channels can provide diverse microhabitats that serve as refugia for the survival of microbes.

## 5 Conclusions

An ExStream manipulation experiment was performed to compare the response of the aquatic bacterial community to a drying perturbation followed by rewetting in streams of different habitats relative to continuous flow using biofilm bacteria as bioindicators. The key finding is that a more heterogeneous habitat does not always support more diverse bacteria. Flow perturbations promoted the specialization of microbial communities. Long-term drying induced a lower richness and more diversity of bacteria with preferences for a fine-mosaic habitat and altered bacterial-associated ecological networks and metabolic functions, with a slightly greater network complexity and vulnerability, and a decline in xylan degraders, nitrogen fixers, and ammonia oxidizers, and an increase in chitin degraders and atrazine metabolizers in streams of diverse-heterogeneity habitats. The drying effects were remediated upon flow resumption through microbial activation and recolonization. This resulted in reduced microbial network complexity and comparable bacterial α-diversity to that in permanent streams, alongside increased chitin and aromatic hydrocarbon degraders in rewetting streams.

This study highlights the resilience of benthic bacteria to hydrological stress. Biofilm bacteria enabled their survival by forming cysts in diverse habitats. Despite species differences across different stream habitats, hydrological connectivity and the functionally analogous species supported by more complex microbial networks contributed to the resilience of benthic bacterial communities and ensured the stability of their ecosystem functions against drought disturbance induced by climate change and anthropogenic disturbances. Therefore, restoring both hydrological connectivity and habitat heterogeneity is essential for enhancing microbial resilience and ecosystem stability in degraded freshwater ecosystems.

## Data Availability

The datasets presented in this study can be found in online repositories. The names of the repository/repositories and accession number(s) can be found below: https://www.ncbi.nlm.nih.gov/, SAMN41274414 - SAMN41274605.
