# Supplementary material for: Functional redundancy enhances microbial resilience in streams: mitigating flow perturbations
Source: Front Microbiol. 2025 Nov 28;16:1581882. doi: 10.3389/fmicb.2025.1581882 (PMC12698528; doi:10.3389/fmicb.2025.1581882)
Supplement: Supplementary file 1 [file Supplementary_file_1.docx]

**Supplementary Material**

**Table S1.** Summary of linear models testing the interactions between habitat and hydrology through time on (a) bacterial α-diversity and (b) abundant bacterial phyla in a mesocosm experiment on the bank of the Yinxi stream, China. P-values in bold where *p* < 0.05. Effect sizes (partial-η^2^ values; range 0-1) are shown in parentheses for all cases where *p* < 0.1 and classified directionally as positive (+) or negative (–); the * indicates a treatment interaction.

(a)

| Response | Habitat | Hydrology | Time | Habitat * Hydrology | Habitat * Time | Hydrology * Time | Habitat * Hydrology * Time |
| --- | --- | --- | --- | --- | --- | --- | --- |
| Bacterial Richness | **0.032**  (–0.040) | **0.026**  (–0.029) | **<0.001** (0.157) | 0.627 | 0.836 | 0.404 | 0.950 |
| Shannon-Wiener Diversity | 0.171 | **<0.001** (+0.073) | **<0.001** (0.678) | 0.964 | 0.101 | **<0.001** (0.113) | 0.810 |

(b)

| Response | Relative Abundance (%) | Habitat | Hydro-  logy | Time | Habitat * Hydrology | Habitat * Time | Hydrology * Time | Habitat * Hydrology * Time |
| --- | --- | --- | --- | --- | --- | --- | --- | --- |
| Proteobacteria | 66.8 | 0.665 | 0.080  (–0.018) | **<0.001**  (0.453) | 0.727 | 0.997 | **0.001**  (0.099) | 0.940 |
| Planctomycetes | 12.63 | **0.011**  (–0.052) | **<0.001**  (–0.099) | **0.012**  (0.063) | 0.633 | 0.387 | 0.353 | 0.699 |
| Bacteroidetes | 4.88 | 0.112 | 0.796 | **<0.001**  (0.667) | 0.999 | 0.917 | 0.517 | 0.943 |
| Verrucomicrobia | 7.01 | 0.291 | **0.020**  (–0.032) | **<0.001**  (0.286) | 0.966 | 0.995 | 0.501 | 0.125 |
| Actinobacteria | 4.85 | 0.075  (–0.030) | 0.409 | 0.165 | 0.716 | 0.198 | 0.573 | 0.401 |
| Gemmatimonadetes | 0.51 | 0.789 | **<0.001**  (+0.342) | **<0.001**  (0.241) | 0.742 | 0.419 | **<0.001**  (0.197) | 0.483 |
| Acidobacteria | 1.17 | 0.258 | 0.456 | **<0.001**  (0.884) | 0.422 | **0.028**  (0.080) | 0.090  (0.038) | 0.688 |
| Firmicutes | 0.06 | 0.633 | **0.001**  (+0.069) | **<0.001**  (0.156) | 0.688 | 0.896 | **<0.001**  (0.157) | 0.871 |
| Armatimonadetes | 0.23 | 0.074  (–0.030) | **<0.001**  (+0.159) | **<0.001**  (0.457) | 0.`380 | 0.744 | **<0.001**  (0.249) | 0.394 |
| Candidate division WPS-1 | 0.15 | **0.013**  (–0.051) | 0.303 | **<0.001**  (0.719) | 0.152 | 0.802 | 0.163 | 0.633 |
| Candidatus Sacchari bacteria | 0.54 | **0.033**  (–0.040) | **<0.001**  (–0.389) | **0.024**  (0.055) | 0.519 | 0.659 | **<0.001**  (0.426) | 0.864 |
| Chloroflexi | 0.1 | 0.214 | **<0.001**  (+0.105) | **<0.001**  (0.728) | 0.327 | **0.008**  (0.097) | **<0.001**  (0.133) | 0.785 |
| Parcubacteria | 0.19 | **0.001**  (–0.081) | **<0.001**  (–0.198) | **0.001**  (0.091) | 0.324 | 0.768 | **<0.001**  (0.213) | 0.599 |
| DeinococcusThermus | 0.22 | 0.128 | **0.019**  (+0.032) | **<0.001**  (0.248) | 0.507 | 0.928 | **<0.001**  (0.139) | 0.888 |

**（c）**

| Treatment | Comparison | Flow Condition | |
| --- | --- | --- | --- |
|  |  | Richness *p* | Shannon *p* |
| Low | 23d-26d | 0.6042 | 0.6446 |
|  | 23d-38d | **0** | **0.0002** |
|  | 23d-61d | 0.0552 | **0** |
|  | 26d-38d | **0.0347** | **0** |
|  | 38d-61d | **0** | **0** |
|  | 23d-26d-38d-61d | **0** | **0** |
| Medium | 23d-26d | 0.0930 | 0.1306 |
|  | 23d-38d | **0** | **0.0028** |
|  | 23d-61d | **0** | **0** |
|  | 26d-38d | **0** | 0.0725 |
|  | 38d-61d | **0** | **0** |
|  | 23d-26d-38d-61d | **0** | **0** |
| High | 23d-26d | 0.6891 | 0.6951 |
|  | 23d-38d | **0** | **0.0002** |
|  | 23d-61d | **0** | **0** |
|  | 26d-38d | **0** | **0.0001** |
|  | 38d-61d | **0** | **0** |
|  | 23d-26d-38d-61d | **0** | **0** |

**Table S2.** Summary of MANOVA results testing the interactions between habitat and hydrology on (a) bacterial α-diversity and (b) abundant bacterial phyla in a mesocosm experiment on the bank of the Yinxi stream, China. P-values in bold where *p* < 0.05. Effect sizes (partial-η^2^ values; range 0-1) are shown in parentheses for all cases where *p* < 0.1 and classified directionally as positive (+) or negative (–); the * indicates a treatment interaction.

| Experiment  period | Habitat | | Hydrology | | Habitat * Hydrology | |
| --- | --- | --- | --- | --- | --- | --- |
|  | Bacterial  Richness | Shannon-Wiener Diversity | Bacterial  Richness | Shannon-Wiener Diversity | Bacterial  Richness | Shannon-Wiener Diversity |
| 23 Day | 0.414 | 0.247 | 0.302 | 0.508 | 0.719 | 0.179 |
| 26 Day | 0.596 | **0.013**  (0.188) | 0.505 | 0.140 | 0.901 | 0.764 |
| 38 Day | 0.068  (–0.120) | 0.290 | **0.021**  (–0.120) | **0.002**  (+0.214) | 0.581 | 0.980 |
| 61 Day | 0.767 | 0.471 | 0.820 | 0.090  (+0.067) | 0.796 | **0.042**  (0.140) |

**Table S3.** Results of the analysis of similarities (ANOSIM with 999 permutations) of testing pair-wise comparisons of biofilm bacterial community composition in the different habitat heterogeneity treatments under: (a) flowing conditions, (b) drying and rewetting conditions, and (c) drying and permanent flowing conditions in a mesocosm experiment on the bank of the Yinxi stream, China. Significant differences (*p* < 0.05) detected after Bonferroni correction in pair-wise comparisons are indicated in bold.

| Colonization period | | Time | Comparison​ | R​ | *p* value |
| --- | --- | --- | --- | --- | --- |
|  | Colonization under flowing conditions | 23-day | Low-Medium​ | 0.01 ​ | 0.32​0 |
|  |  |  | Low-High​ | 0.07​ | **0.04​0** |
|  |  |  | Medium-High​ | 0.02​ | 0.24​0 |
|  |  |  | Low-Medium​-High | 0.03 | 0.110 |
|  |  | 26-day | Low-Medium​ | 0.07 | 0.140 |
|  |  |  | Low-High​ | -0.01 | 0.530 |
|  |  |  | Medium-High​ | -0.04 | 0.670 |
|  |  |  | Low-Medium​-High | 0.01 | 0.360 |
|  |  | 38-day | Low-Medium​ | -0.08 | 0.880 |
|  |  |  | Low-High​ | 0.06 | 0.180 |
|  |  |  | Medium-High​ | -0.01 | 0.460 |
|  |  |  | Low-Medium​-High | -0.01 | 0.530 |
|  |  | 61-day | Low-Medium​ | -0.02 | 0.490 |
|  |  |  | Low-High​ | 0.19 | **0.020** |
|  |  |  | Medium-High​ | 0.04 | 0.240 |
|  |  |  | Low-Medium​-High | 0.06 | 0.130 |
| (b) | Drying & rewetting conditions | Treatment | Comparison​ | R | *p* value |
|  |  | 3-day Drying | Low-Medium​ | 0.04 | 0.260 |
|  |  |  | Low-High​ | 0.04 | 0.240 |
|  |  |  | Medium-High​ | -0.00 | 0.460 |
|  |  |  | Low-Medium​-High | 0.003 | 0.260 |
|  |  | 15-day Drying | Low-Medium​ | -0.06 | 0.850 |
|  |  |  | Low-High​ | 0.02 | 0.320 |
|  |  |  | Medium-High​ | 0.10 | 0.060 |
|  |  |  | Low-Medium​-High | 0.02 | 0.270 |
|  |  | Rewetting | Low-Medium​ | -0.02 | 0.540 |
|  |  |  | Low-High​ | 0.04 | 0.260 |
|  |  |  | Medium-High​ | 0.02 | 0.360 |
|  |  |  | Low-Medium​-High | 0.02 | 0.290 |
| (c) | Between drying/rewetting and permanent flowing conditions | Treatment | Comparison​ | R​ | *p* value |
|  |  | High | 3d Drying - 26d | 0.39 | **0.001** |
|  |  |  | 15d Drying - 38d​ | 0.62 | **0.001** |
|  |  |  | Rewetting - 61d | 0.67 | **0.001** |
|  |  | Medium | 3d Drying - 26d | 0.62 | **0.001** |
|  |  |  | 15dDrying - 38d​ | 0.73 | **0.001** |
|  |  |  | Rewetting - 61d | 0.78 | **0.001** |
|  |  | Low | 3d Drying - 26d | 0.36 | **0.001** |
|  |  |  | 15d Drying - 38d​ | 0.59 | **0.001** |
|  |  |  | Rewetting - 61d | 0.85 | **0.001** |

**Table S4.** Results (*p*-values and partial-η^2^ effect sizes) from the MANOVA testing the interactions between habitat and hydrology on the 14 most-abundant bacterial phyla after: (a) three days drying, (b) 15 days drying and (c) rewetting conditions in a mesocosm experiment on the bank of the Yinxi stream, China. P-values in bold font where *p* < 0.05. Effect sizes (partial-η^2^ values; range 0-1) are shown in parentheses for all cases where *p* < 0.1 and classified directionally as positive (+) or negative (–); the * indicates a treatment interaction.

| Bacterial Phyla | 1. 3-day Drying | | | 1. 15-day Drying | | | | 1. 23-day Rewetting | | | | |
| --- | --- | --- | --- | --- | --- | --- | --- | --- | --- | --- | --- | --- |
|  | Habitat | Hydrology | Habitat * Hydrology | | Habitat | Hydrology | Habitat * Hydrology | | Habitat | Hydrology | Habitat * Hydrology |  |
| Proteobacteria | 0.704  (0.017) | 0.576  (0.007) | 0.456  (0.037) | | 0.912  (0.004) | **<0.001**  (–0.290) | 0.999  (0) | | 0.747  (0.014) | 0.166  (0.045) | 0.491  (0.033) |  |
| Planctomycetes | 0.707  (0.016) | **0.084**  (–0.070) | 0.451  (0.037) | | **0.035**  (–0.147) | **0.005**  (–0.174) | 0.479  (0.034) | | 0.814  (0.010) | **0.024**  (–0.115) | 0.895  (0.005) |  |
| Bacteroidetes | **0.050**  (–0.133) | 0.515  (0.010) | 0.906  (0.005) | | 0.810  (0.010) | 0.364  (0.020) | 0.771  (0.012) | | 0.859  (0.007) | 0.505  (0.011) | 0.808  (0.010) |  |
| Verrucomicrobia | 0.695  (0.017) | **0.065**  (–0.079) | 0.122  (0.095) | | 0.823  (0.009) | 0.624  (0.006) | 0.179  (0.079) | | 0.312  (0.054) | **0.018**  (–0.126) | 0.751  (0.014) |  |
| Actinobacteria | 0.561  (0.027) | 0.108  (0.060) | 0.683  (0.018) | | **0.085**  (–0.111) | 0.676  (0.004) | 0.240  (0.066) | | 0.625  (0.022) | 0.323  (0.023) | 0.656  (0.020) |  |
| Gemmatimonadetes | 0.884  (0.006) | **<0.001**  (+0.438) | 0.532  (0.030) | | 0.773  (0.012) | **<0.001**  (+0.419) | 0.635  (0.021) | | **0.021**  (–0.169) | **<0.001**  (+0.533) | 0.109  (0.100) |  |
| Acidobacteria | **0.054**  (+0.130) | **0.086**  (–0.068) | 0.939  (0.003) | | **0.017**  (+0.177) | **0.036**  (+0.100) | 0.563  (0.027) | | 0.309  (0.054) | 0.393  (0.017) | 0.422  (0.040) |  |
| Armatimonadetes | 0.151  (0.086) | **<0.001**  (+0.546) | 0.654  (0.020) | | 0.796  (0.011) | **0.001**  (+0.249) | 0.356  (0.048) | | 0.426  (0.040) | 0.830  (0.001) | 0.532  (0.030) |  |
| Candidatus Sacchari bacteria | 0.172  (0.080) | **0.001**  (–0.221) | 0.682  (0.018) | | 0.178  (0.079) | **<0.001**  (–0.767) | 0.723  (0.015) | | 0.744  (0.014) | **0.005**  (–0.174) | 0.321  (0.053) |  |
| Firmicutes | 0.653  (0.020) | **0.014**  (+0.134) | 0.986  (0.001) | | 0.678  (0.018) | **0.002**  (+0.206) | 0.676  (0.018) | | 0.316  (0.053) | **<0.001**  (+0.306) | **0.022**  (–0.166) |  |
| Chloroflexi | 0.346  (0.049) | 0.948  (0) | 0.800  (0.011) | | **0.017**  (–0.175) | **<0.001**  (+0.328) | 0.348  (0.049) | | 0.300  (0.056) | **0.001**  (+0.222) | 0.613  (0.023) |  |
| Candidate division WPS-1 | 0.179  (0.079) | 0.900  (0) | 0.208  (0.072) | | **0.020**  (+0.171) | 0.393  (0.017) | 0.768  (0.012) | | 0.495  (0.033) | 0.158  (0.047) | 0.339  (0.050) |  |
| Parcubacteria | **0.098**  (–0.105) | 0.692  (0.004) | 0.662  (0.019) | | 0.162  (0.083) | **<0.001**  (–0.357) | 0.358  (0.048) | | 0.139  (0.090) | **<0.001**  (–0.459) | 0.622  (0.022) |  |
| Deinococcus-Thermus | 0.834  (0.009) | **0.054**  (–0.085) | 0.582  (0.025) | | 0.960  (0.002) | 0.120  (0.057) | 0.585  (0.025) | | 0.119  (0.096) | **<0.001**  (+0.424) | 0.622  (0.022) |  |

**Table S5.** Topological properties of their associated empirical networks of bacterial community. AD average degree, avgCC average clustering coefficient, GD graph density.

|  | Colonization | 15-day Control | 15-day Dry | Rewetting Control | Rewetting |
| --- | --- | --- | --- | --- | --- |
| Total nodes | 233 | 536 | 930 | 818 | 781 |
| Total edges | 457 | 2412 | 13764 | 2434 | 1163 |
| Modules | 26 | 26 | 24 | 82 | 91 |
| Module hubs | 14 | 15 | 17 | 14 | 16 |
| Connectors | 11 | 15 | 15 | 11 | 8 |
| R^2^ of Power-law | 0.749 | 0.540 | 0.262 | 0.901 | 0.901 |
| Connectance | 0.017 | 0.017 | 0.032 | 0.007 | 0.004 |
| AD | 3.922 | 9 | 29.6 | 5.95 | 2.978 |
| avgCC | 0.315 | 0.378 | 0.445 | 0.426 | 0.239 |
| GD | 0.017 | 0.017 | 0.032 | 0.007 | 0.004 |
| Modularity | 0.665 | 0.61 | 0.528 | 0.627 | 0.804 |
| Positive proportion | 0.989 | 0.964 | 0.968 | 0.921 | 0.926 |
| Robustness | 0.993 | 0.99765 | 0.9988 | 0.998 | 0.997 |
| Vulnerability | 0.371 | 0.9195 | 0.973 | 0.802 | 0.769 |
| Complexity | 0.394 | 0.570 | 0.676 | 0.379 | 0.222 |

**Table S6.** Results (p-values and partial-η2 effect sizes) of the MANOVA (multivariate and univariate results) on the 11 most-abundant bacterial metabolic functional groups after: (a) three days drying, (b) 15 days drying and (c) under rewetting conditions in a mesocosm experiment on the bank of the Yinxi stream, China. P-values in bold font where *p* < 0.05. Effect sizes (partial-η2 values; range 0-1) are shown in parentheses for all cases where *p* < 0.1 and classified directionally as positive (+) or negative (–); the * indicates a treatment interaction.

| Bacterial metabolic  functional groups | 1. 3-day Drying | | | 1. 15-day Drying | | | | 1. 23-day Rewetting | | | | |
| --- | --- | --- | --- | --- | --- | --- | --- | --- | --- | --- | --- | --- |
|  | Habitat | Hydrology | Habitat * Hydrology | | Habitat | Hydrology | Habitat * Hydrology | | Habitat | Hydrology | Habitat * Hydrology |  |
| Sulfate reducer | 0.512  (0.355) | 0.990  (0) | 0.716  (0.016) | | 0.515  (0.031) | **<0.001**  (–0.260) | 0.749  (0.014) | | 0.584  (0.025) | 0.165  (0.045) | 0.794  (0.011) |  |
| Ammonia oxidizer | 0.583  (0.025) | 0.391  (0.018) | 0.706  (0.016) | | 0.997  (0) | **0.059**  (–0.082) | 0.941  (0.003) | | 0.708  (0.016) | 0.130  (0.054) | 0.971  (0.001) |  |
| Dehalogenator | 0.823  (0.009) | **0.005**  (–0.176) | 0.808  (0.010) | | 0.748  (0.014) | 0.764  (0.002) | 0.961  (0.002) | | 0484  (0.034) | 0.180  (0.042) | 0.729  (0.015) |  |
| Nitrite reducer | 0.350  (0.049) | **0.056**  (+0.084) | 0.432  (0.039) | | 0.834  (0.009) | 0.571  (0.008) | 0.988  (0.001) | | 0.265  (0.061) | 0.166  (0.045) | 0.882  (0.006) |  |
| Sulfide oxidizer | 0.798  (0.011) | 0.204  (0.038) | 0.437  (0.039) | | 0.354  (0.048) | 0.119  (0.057) | 0.933  (0.003) | | 0.450  (0.037) | 0.256  (0.031) | 0.336  (0.051) |  |
| Xylan degrader | 0.360  (0.047) | **0.047**  (–0.091) | 0.544  (0.029) | | **0.013**  (–0.188) | **0.097**  (–0.064) | 0.484  (0.034) | | 0.968  (0.022) | **0.085**  (–0.069) | 0.969  (0.002) |  |
| Nitrogen fixer | 0.892  (0.005) | 0.423  (0.015) | 0.350  (0.049) | | 0.321  (0.053) | **<0.001**  (–0.319) | 0.726  (0.015) | | 0.736  (0.014) | 0.774  (0.002) | 0.532  (0.030) |  |
| Aromatic hydrocarbon degrader | **0.017**  (– 0.178) | 0.143  (0.050) | 0.451  (0.037) | | **0.020**  (–0.169) | 0.573  (0.008) | 0.227  (0.068) | | 0.344  (0.050) | **0.009**  (+0.153) | 0.535  (0.029) |  |
| Chitin degrader | 0.314  (0.054) | 0.808  (0.001) | 0.927  (0.004) | | 0.525  (0.030) | **0.010**  (+0.146) | 0.885  (0.006) | | 0.729  (0.015) | **0.001**  (+0.243) | 0.365  (0.047) |  |
| Sulfur oxidizer | 0.311  (0.054) | **0.015**  (–0.132) | 0.897  (0.005) | | **0.013**  (–0.186) | 0.488  (0.012) | 0.712  (0.016) | | 0.540  (0.029) | 0.101  (0.063) | 0.852  (0.008) |  |
| Atrazine metabolizer | 0.962  (0.002) | 0.842  (0.001) | 0.480  (0.034) | | 0.433  (0.039) | **0.007**  (+0.159) | 0.747  (0.014) | | 0.879  (0.006) | 0.372  (0.019) | **0.021**  (+0.169) |  |

**Figure S1.** Conceptual overview of the *Ex-Stream System* mesocosm experiment set up on the bank of the Yinxi stream, China. After a 23-day initial colonization period, the bacterial community was subjected to two flow treatments: 38 days of continuous flow (Control treatment), versus a 15-day drought followed by a 23-day rewetting period (Drying treatment). These two flow treatments were fully crossed with the three habitat heterogeneity treatments (Low, Medium, High), which were implemented from the start of the colonization period.
